# Liushen Capsules, a promising clinical candidate for COVID-19, alleviates SARS-CoV-2-induced pulmonary in vivo and inhibits the proliferation of the variant virus strains in vitro

**DOI:** 10.1186/s13020-022-00598-4

**Published:** 2022-04-01

**Authors:** Qinhai Ma, Biao Lei, Ruihan Chen, Bin Liu, Wencong Lu, Haiming Jiang, Zexing Chen, Xiaowen Guo, Yutao Wang, Lu Zhang, Qiaoyan Chen, Xiaobo Li, Zifeng Yang

**Affiliations:** 1 State Key Laboratory of Respiratory Disease, National Clinical Research Center for Respiratory Disease, Guangzhou Institute of Respiratory Health, The First Affiliated Hospital of Guangzhou Medical University, Guangzhou, Guangdong People’s Republic of China; 2 Guangzhou Laboratory, Guangzhou, Guangdong People’s Republic of China; 3 Guangdong Women and Children Hospital, Guangzhou, Guangdong People’s Republic of China; 4 Technology Centre, Guangzhou Customs, Guangzhou, People’s Republic of China; 5 Guangdong Provincial Hospital of Chinese Medicine, The Second Affiliated Hospital, Guangzhou University of Chinese Medicine, Guangzhou, Guangdong People’s Republic of China

**Keywords:** Liushen Capsules, SARS-CoV-2, COVID-19, The 501Y.V2/B.1.35 and G/478K.V1/ B.1.617.2 strains, Pulmonary

## Abstract

**Background:**

Coronavirus disease 2019 (COVID-19) causes a global pandemic and has devastating effects around the world, however, there are no specific antiviral drugs and vaccines for the constant mutation of SARS-CoV-2.

**Purpose:**

In this study, we evaluted the antiviral and anti-inflammatory activities of Liushen Capsules (LS) on different novel coronavirus in vitro, studied its therapeutic effects on novel SARS-CoV-2 infected mice and observed the LS’s clinical efficacy and safety in COVID-19.

**Methods:**

The antiviral and aiti-inflammatory effects of LS on the 501Y.V2/B.1.35 and G/478K.V1/ B.1.617.2 strains were determined in vitro. A hACE2 mouse model of novel SARS-CoV-2 pneumonia was established. Survival rates, histological changes, inflammatory markers, lung virus titers and the expression of the key proteins in the NF-κB/MAPK signaling pathway was detected by western blotting and immumohistochemical staining in the lungs were measured. Subsequently, the disease duration, prognosis of disease, time of negative nucleic acid and the cytokines levels in serum were used to assess the efficacy of treatment with LS in patients.

**Results:**

The results showed that LS (2, 1, 0.5 μg/mL) could significantly inhibit the replication of the two SARS-CoV-2 variants and the expression of pro-inflammatory cytokines (IL-6, IL-8, IP-10, CCL-5, MIP-1α, IL-1α) induced by the virus in vitro. As for the survival experiment in mice, the survival rate of virus group was 20%, while LS-treatment groups (40, 80, 160 mg/kg) could increase the survival rate to 60, 100 and 100%, respectively. LS (40, 80, 160 mg/kg) could significantly decrease the lung titers in mice and it could improve the pathological changes, inhibit the excessive inflammatory mediators (IFN-α, IFN-γ, IP-10, MCP-1) and the protein expression of p-NF-κB p65 in mice. Moreover, LS could significantly decrease SARS-CoV-2-induced activation of p-NF-κB p65, p-IκBα, and p-p38 MAPK and increase the protein expression of the IκBα. In addition, the patient got complete relief of symptoms after being treated with LS for 6 days and was proven with negative PCR test after being treated for 23 days. Finally, treatment with LS could reduce the release of inflammatory cytokines (IL-6, PDGF-AA/BB, Eotaxin, MCP-1, MIP-1α, MIP-1β, GRO, CCL-5, MCP-3, IP-10, IL-1α).

**Conclusion:**

LS effectively alleviated novel SARS-CoV-2 or variants induced pneumonia in vitro and in vivo, and improved the prognosis of COVID-19. In light of the efficacy and safety profiles, LS could be considered for the treatment of COVID-19 with a broad-spectrum antiviral and anti-inflammatory agent.

## Introduction

Coronavirus disease 2019 (COVID-19), caused by severe acute respiratory syndrome coronavirus 2 (SARS-CoV-2), has led to a global pandemic and had devastating effects around the world. As of 26 September 2021, the ongoing pandemic of COVID-19 had resulted in 231,703,120 confirmed cases and 4,746,620 confirmed deaths worldwide. Patients with confirmed SARS-CoV-2 infection were presented with clinical spectrum from asymptomatic infection to critical illness. Chinese epidemiological data show that COVID-19 with mild or moderate disease (including patients without pneumonia and with mild pneumonia) account for 81% and the percentage of patients with severe disease and critical illness were 14% and 5%, respectively [1]. Patients ranging from moderate to critical illness are required to be closely monitored, even hospitalized and accepted further treatment. Patients with risk factors including older age, cardiovascular, diabetes, obesity and chronic lung disease tend to merge COVID-19.

SARS-CoV-2 viriants have caused a pandemic around the world, which have posed an increase risk on public health. At present, there are four SARS-CoV-2 variants, including GR/501Y.V1/B.1.1.7, GH/501Y.V2/B.1.351, GR/501Y.V3/P.1 and G/478K.V1/B.1.617.2, which are considered as the variants of concern (VOC) by WHO for their higher hospitalization risks, rapid spread and decrease in effectiveness of neutralizing antibodies and some vaccines [2]. Of note, mutations appearing in immunodominant domains of the spike protein such as the 501Y.V2/B.1.35 strain can result in escaping from neutralizing antibodies in COVID-19 convalescent plasma and ineffectiveness of directed monoclonal antibodies and vaccines, which highlight the prospect of reinfection and an decrease in effectiveness of current spike-based vaccines [3]. Thus, it’s urgent to find more effective strategies for prevention and treatment of COVID-19 caused by mutant strains, and it’s of great benefit to early access to the antiviral therapy before or soon after symptom onset [4]. Several medications have been proposed as treatment for COVID-19 by WHO including Hydroxychloroquine, Lopinavir-ritonavir, Remdesivir, Corticosteroids [5]. However, antiviral medications are also faced with limitations. It was reported that lopinavir/ritonavir or arbidol was not effective in adult patients with mild/moderate disease [6] and that Hydroxychloroquine [7] and Remdesivir [8] did not significantly improve the clinical outcome with moderate COVID-19. Thus, effective antiviral strategies are urgently needed.

Traditional Chinese Medicine (TCM) is extensively used in the treatment of life-threatening respiratory viral infection including SARS-CoV, MERS-CoV, dengue virus, and influenza A virus (including H1N1 and H7N9). According to the transcriptional study and some preclinical experiments, the mechanism of TCM on SARS-CoV-2 mainly include targeting SARS-CoV-2, reducing pro-inflammatory cytokines, protecting multiple organs from damage and preventing lung fibrosis [9, 10]. During the epidemic of SARS-CoV-2 in China, TCM were recommended for the treatment of COVID-19 by Chinese government and got favorable clinical outcome. Mild, moderate, and even critical COVID-19 has been benefited from treatment with TCM. Several study indicated that TCM, such as Lianhuaqingwen [11], Hanshiyi Formula [12], ReDuNing injection [13] and Qingfei Paidu decoction [14], could effectively ameliorate symptoms (including fever, cough and fatigue), shorten the duration of viral shedding and decrease the excessive release of cytokines in mild and moderate COVID-19. Xuebijing could improve lung injury in patients with severe or critical COVID-19 [15]. Keguan-1-based integrative therapy was safe and superior to the standard therapy in suppressing the development of acute respiratry distress syndrme (ARDS) in COVID-19 patients [16].

Liushen Capsules (LS), a TCM, exhibits multiple biological activities, including anti-viral, antibacterial, anti-inflammatory, anti-tumor and immunomodulatory activities, which has been used for treating viral infection, cancer, heart disease and so on. Our previous study had indicated that LS displayed inhibitory activities against influenza A/PR/8(H1N1) and markedly ameliorated lung inflammation and injury in mice caused by influenza A/PR/8(H1N1) via TLR4/NF-кB signaling pathway [27]. In addition, LS prevented S. aureus adherence to influenza virus-infected A549 cells by downregulating the expression of the adhesion molecule CEACAM1 in vitro and significantly alleviated lung injury induced by viral and secondary bacterial infection [28]. Furthermore, our previous research has found that LS had antiviral characteristic towards novel SARS-CoV-2 and significantly reduced the excessive release of inflammatory mediators induced by SARS-CoV-2 in vitro [17], which indicated that LS had the potential to be a treatment for COVID-19. However, the efficacy of LS on COVID-19 patients, SARS-CoV-2 pneumonia mice and the 501Y.V2/B.1.35 and G/478K.V1/B.1.617.2 strain remains obscure. In this study, we aimed to verified its inhibitory effect on the 501Y.V2/B.1.35 and G/478K.V1/B.1.617.2 strains in vitro, determine the protective effect in mice infected with SARS-CoV-2, and observe the efficacy of LS in COVID-19 patients, which may result in gaining a comprehensive understanding of the treatment of LS against COVID-19.

## Methods

### Reagents

LS (lot: SA01004C) was provided by Suzhou Leiyunshang Medicine Pharmaceutical Co., Ltd. (Suzhou, China). In the previous study, the index components in LS were detected by high-performance liquid chromatography (HPLC). HPLC revealed that LS contained 0.12% gamabufotalin, 0.10% arenobufagin, 0.26% telocinobufagin, 0.21% desacetylcinobufotalin, 0.25% bufotalin, 0.41% cinobufotalin, 0.27% bufalin, 0.70% resibufogenin, 0.68% cinobufagin, 1.81% cholic acid, 0.27% anserine deoxycholic acid, and 0.23% deoxycholic acid [18].

### Cells, Viral strains and Animals

African green monkey kidney epithelial (Vero E6) cells were purchased from ATCC,which were cultured in Dulbecco’s modified Eagle’s medium (DMEM, Gibco, USA) with 10% fetal bovine serum (FBS), 100 U/mL penicillin, and 100 μg/mL streptomycin. The novel coronavirus (SARS-CoV-2) (50% tissue culture infective dose (TCID_50_) = 10^–7^/100 μL) were clinically isolated from the First Affiliated Hospital of Guangzhou Medical University. The G/478K.V1/B.1.617.2 strain (TCID_50_ = 10^–6.5^/100 μL)and the 501Y.V2/B.1.35 strain (TCID_50_ = 10^–6^/100 μL) were clinically isolated from Guangzhou City Eighth People's Hospital. The virus was propagated and adapted as previously described [19]. The TCID_50_ of SARS-CoV-2 were determined using the Reed–Muench method. Virus stocks were collected and stored at − 80 °C. Pathogen-free K18-hACE2 C57BL/6 mice (18–22 g, SPF, Certificate No. SCXK (Su) 2018-0008) were purchased from Jiangsu Jicui Yaokang Biotechnology Co., Ltd.

### Cytopathic effect (CPE) inhibition assay

To determine the antiviral effects of LS against the 501Y.V2/B.1.35 and G/478K.V1/B.1.617.2 strains, the CPE inhibition assay under the nontoxic concentration of LS was employed. The Vero E6 cell monolayers (5 × 10^4^ cells/well) were seeded in 96-well plates and inoculated with 100 TCID_50_ of the virus at 37 °C for 2 h. The inoculum was removed, and the cells were subsequently incubated with indicated concentrations of LS or the positive control remdesivir. Following 72 h of incubation, the percentage of CPE in LS-treated cells was recorded. The 50% inhibition concentration (IC_50_) of the virus-induced CPE by LS was calculated as described and the selectivity index (SI) was determined from the TC_50_ to IC_50_ ratio [20].

### Plaque reduction assay

The plaque reduction assay was performed as previously described [17]. Briefly, Vero E6 cells (2 × 10^6^ cells/well) were grown in a 12 well plate at 37 °C for 24 h. The monolayer of cells was washed with phosphate buffered saline (PBS) and infected at MOI of 1 of the 501Y.V2/B.1.35 strain or the G/478K.V1/ B.1.617.2 strain. After incubation for 2 h, the viral suspension was removed and overlaid with 0.8% agarose in medium with or without the indicated dose of LS and remdesivir for 72 h. Subsequently, the cells were fixed by 4% formalin for 30 min and stained with 1% crystal violet for 5 min. The number of plaque was counted.

### Quantitative RT-PCR assay

The cell monolayer (5 × 10^5^ cells/well) in 12-well plates were washed with PBS and then were inoculated with the 501Y.V2/B.1.35 strain or G/478K.V1/ B.1.617.2 strain at MOI of 1 for 2 h. The inoculums were removed after infection, and the cells were divided into six groups: normal control group (NC), virus-infected group (virus), positive control group (remdesivir), three concentrations of LS (2, 1, 0.5 μg/mL), the cells were harvested at 48 h. Total RNA from the different groups was extracted according to the specification of RNA reagent (Invitrogen, MA, USA), and reverse transcription of RNAs was quantified by using the PrimeScript™ RT Master Mix kit (Takara Bio, Japan). Then, RT-PCR was performed on cDNA samples via the SYBR Premix Ex Tap™ II (Takara Bio, Japan). The PCR data were analyzed using the detection system (ABI PRISM® 7500 Real-time PCR system, Applied Biosystems Co., USA). The relative amount of PCR products was calculated using the 2^−ΔΔCt^ method as previously described [21].

### Acute lung injury experiment

A total of 30 hACE2 transgenic mice were randomly divided into six groups (n = 5 per group). The normal group was intranasally administered with 50 μL PBS without SARS-CoV-2 and other groups were intranasally inoculated with 50 µL of viral suspension. The infected mice were administrated LS (160, 80 or 40 mg/kg), remdesivir (50 mg/kg), or PBS daily for 5 days by gavage after infection for 2 h. The symptoms and mortality of the mice were observed for 5 days. After infection for 5 days, the lung tissues were used to determine the lung virus titers, lung inflammatory factors and histopathology. This animal experiment was approved by the Ethics Committee of Guangzhou Medical University (2021061).

### Determination of lung virus titers

Lung tissues of acute lung injury experiment were homogenized in 1 mL PBS. The supernatants of homogenized lung were serially tenfold diluted and inoculated in corresponding wells of Vero E6 cells. The 50% tissue culture infective dose (TCID_50_) was used to determine the lung virus titers.

### Determination of lung inflammatory factors

The primers of IL-6, IL-8, IP-10, CCL-5, MIP-1α, IL-1α, IFN-α, IFN-γ, MCP-1 and GAPDH genes (Table 1) were designed by using Primer 5.0. The lung tissues of acute lung injury experiment were homogenized as it mentioned above. The total RNA of the supernatants was extracted according to the specification of RNA reagent (Invitrogen, MA, USA), and reverse transcription of RNAs was quantified by using the PrimeScript™ RT Master Mix kit (Takara Bio, Japan). Then, RT-PCR was performed on cDNA samples via the SYBR Premix Ex Tap™ II (Takara Bio, Japan). The PCR data were analyzed by using the detection system (ABI PRISM® 7500 Real-time PCR system, Applied Biosystems Co., USA). The relative amount of PCR products was calculated using the 2^−∆∆Ct^ method as previously described [21].

**Table 1:** Primer sequence for RT-qPCR

Target gene	Direction	Sequence (5′-3′)
IFN-α (mouse)	Fwd	ACTCATTCTGCACTGGCCTCCA
	Rev	ACTTCTGCTCTGACCACCTCCC
IFN-γ (mouse)	Fwd	TGACATGAAAATCCTGCAGAGCC
	Rev	GCTGGACCTGTGGGTTGTTGAC
IP-10 (mouse)	Fwd	TGAGGGCCATAGGGAAGCTTGAAAT
	Rev	TCCGGATTCAGACATCTCTGCTCAT
MCP-1 (mouse)	Fwd	AAGATCTCAGTGCAGAGGCTCG
	Rev	CCA GGGGTAGAACTGTGGTTCAA
GAPDH(mouse)	Fwd	CAAAATGGTGAAGGTCGGTGTG
	Rev	GTTGAGGTCAATGAAGGGGTCG
IL-6 (human)	Forward	CGGGAACGAAAGAGAAGCTCTA
	Reverse	CGCTTGTGGAGAAGGAGTTCA
CCL-5 (human)	Forward	CAGCAGTCGTCTTTGTCACC
	Reverse	GTTGATGTACTCCCGAACCC
IL-8 (human)	Forward	CTTGGTTTCTCCTTTATTTCTA
	Reverse	GCACAAATATTTGATGCTTAA
IP-10 (human)	Forward	GAAATTATTCCTGCAAGCCAATTT
	Reverse	TCACCCTTCTTTTTCATTGTAGCA
IL-1α (human)	Forward	GAAGATGTGCCTGTCCTGTGT
	Reverse	CGCTCAGGTCAGTGATGTTAA
MIP-1α (human)	Forward	CTGCATCACTTGCTGCTGACA
	Reverse	CACTGGCTGCTCGTCTCAAAG
GAPDH(human)	Forward	GAAGGTGAAGGTCGGAGTC
	Reverse	GAAGATGGTGATGGGATTTC

### Histopathology

One of the lungs of acute lung injury experiment was removed and fixed immediately in 4% paraformaldehyde and used for histological evaluation. The tissue fixed in 4% paraformaldehyde was dehydrated and embedded in paraffin and sections were mounted on glass slide and stained with H&E to evaluate the severity of pneumonia (Table 2).

**Table 2:** Protein expression of inflammatory factors in patients on day 1 and day 7

inflammatory meditors (pg/ml)	Patient A		Patient B	
	Day1	Day7	Day1	Day7
PDGF-AA/BB	417.63	2018.76	2390.86	1662.27
Eotaxin	19.05	83.34	41.54	22.47
MCP-1	15.3	345.62	45.36	28.24
MIP-1α	0.75	85.26	7.02	4.38
MIP-1β	52.65	343.41	250.35	227.16
GRO	103.05	716.22	467.43	536.83
CCL-5	4264.44	33,165.25	24,171.29	17,321.52
MCP-3	0.31	55.25	0.86	1.32
IL-6	1.32	53.78	7.28	1.45
IP-10	113.95	468.62	478.84	311.53
IL-1α	55.53	55.53	308.72	143.43

### Immunohistochemistry

The lung was fixed with formaldehyde and blocked with 10% BSA (Bovine serum albumin) for 90 min at room temperature. Lung Sects. (5 μm thickness) were incubated at 4 °C with primary antibody against p-NF-κB p65 (1:100, abcam, USA) for 24 h followed by a 15 min wash with PBS. After that, the slides were incubated with a goat anti-rabbit IgG H&L (HRP) at 1:250 for 60 min at room temperature. After being incubated with 3,3’-diaminobenzidine (DAB) and hydrogen peroxide, the slides were visualized with a fluorescence microscope (Zeiss Axiovert 135, Zeiss, Oberkochen, Germany) and assessed by Image-Pro Plus 6.0.

### Western blot assay

The total proteins of the samples were extracted from the cells with radioimmunoprecipitation assay (RIPA) buffer (DGCS Biotechnology, China). The protein concentrations of the samples were detected by using the BCA kit (Beyotime, China). Then, 30 mg of the cell extract was separated by 8% sodium dodecyl sulfate polyacrylamide gel electrophoresis (SDS-PAGE), and then they were transferred to a polyvinylidene fluoride (PVDF) membranes (Millipore, USA). The membranes were blocked with 5% BSA and incubated with different primary antibodies over night at 4 °C. And then, the membranes with different primary antibodies were incubated with different secondary antibodies for 1 h. The immune complexes were immunoblotted and the immunodetection was performed by using the enhanced chemiluminescence reagents (Fdbio, China).

### Patients

Two hospitalized adult patients with PCR-proven SARS-CoV-2 infection in the First Affiliated Hospital of Guangzhou Medical University were included. This study was approved by the Ethics Committee of the First Affiliated Hospital of Guangzhou Medical University and conducted in accordance with GCP guidelines and the Declaration of Helsinki. Written informed consent was obtained from the patients or legal representatives, and the study was registered with the requisite authority (number, 2020054; Guangzhou, China).

Patient A, a 54-year-old man, was included On March 17, 2020. Patient B, a 50-year-old man, was included On March 28.

Patients were recruited after satisfying the criteria: (1) age ≥ 18 years; (2) axillary temperature ≥ 37.3 °C; (3) mild, normal and severe patients with positive SARS-CoV-2 test; (4) Granting of written informed consent.

Patients were excluded if (1) Severe patients satisfying the following situation: I. Respiratory failure with mechanical ventilation; II. shock; III. Combined with organ failure accepting ICU monitoring and treatment; (2) Combined with other respiratory virus infections; (3) Accepted LS treatment with confirmed SARS-CoV-2 infection in a month; (4) Patients allergic to the study drug or patients with allergies; (5) Lactating or pregnant women, women with positive urine pregnancy test or disagree with contraception after participating in the trial for three months; (6) Immunosuppressed patients: malignant tumor; AIDS; having taken immune inhibitors during the last 3 months, having had an organ or bone-marrow transplant; (7) People with severe liver damage and kidney damage; (8) Patients who gave up rescue; (9) Other patients who were not suitable to participate in this study.

### Study treatment

We randomly assigned participants to receive either basic treatment or LS (0.19 g three times a day), Arbidol Hydrochloride (200 mg three times a day) and basic treatment.

Patient A was intervened with basic treatment, while Patient B was treated with LS (0.19 g three times a day), Arbidol Hydrochloride (200 mg three times a day) and basic treatment.

### Proinflammatory cytokine detection in the human serum

Multiple cytokines in serum such as MCP-1, MIP-1β, IL-6, IP-10, sCD40L and so on were monitored. The serum samples were measured by a Bio-Plex Pro Human Cytokine Screening Panel (Bio-Rad, USA). Sample size was estimated based on the above primary outcome measures. A Bio-Plex Luminex 200 XYP instrument (Bio-Rad Laboratories, USA) were used to analyze the assay plate.

### Statistical analysis

Results were presented as means ± standard deviation (S.D.). Data were analyzed using analysis of variance (ANOVA) with SPSS ver. 19.0 (IBM Corp., Armonk, NY, USA). Differences between the groups were determined by one-way ANOVA followed Tukey’s honest significant difference (HSD) test. The Wilcoxon rank-sum test was used for data that did not conform to normal distribution. Count data were presented as percentage values, and comparisons between groups were performed using a χ2 test. Differences were considered significant at P < 0.05.

## Results

### LS exerts antiviral effects on the 501Y.V2/B.1.35 strain and G/478K.V1/ B.1.617.2 strain in vitro

**Fig. 1:**
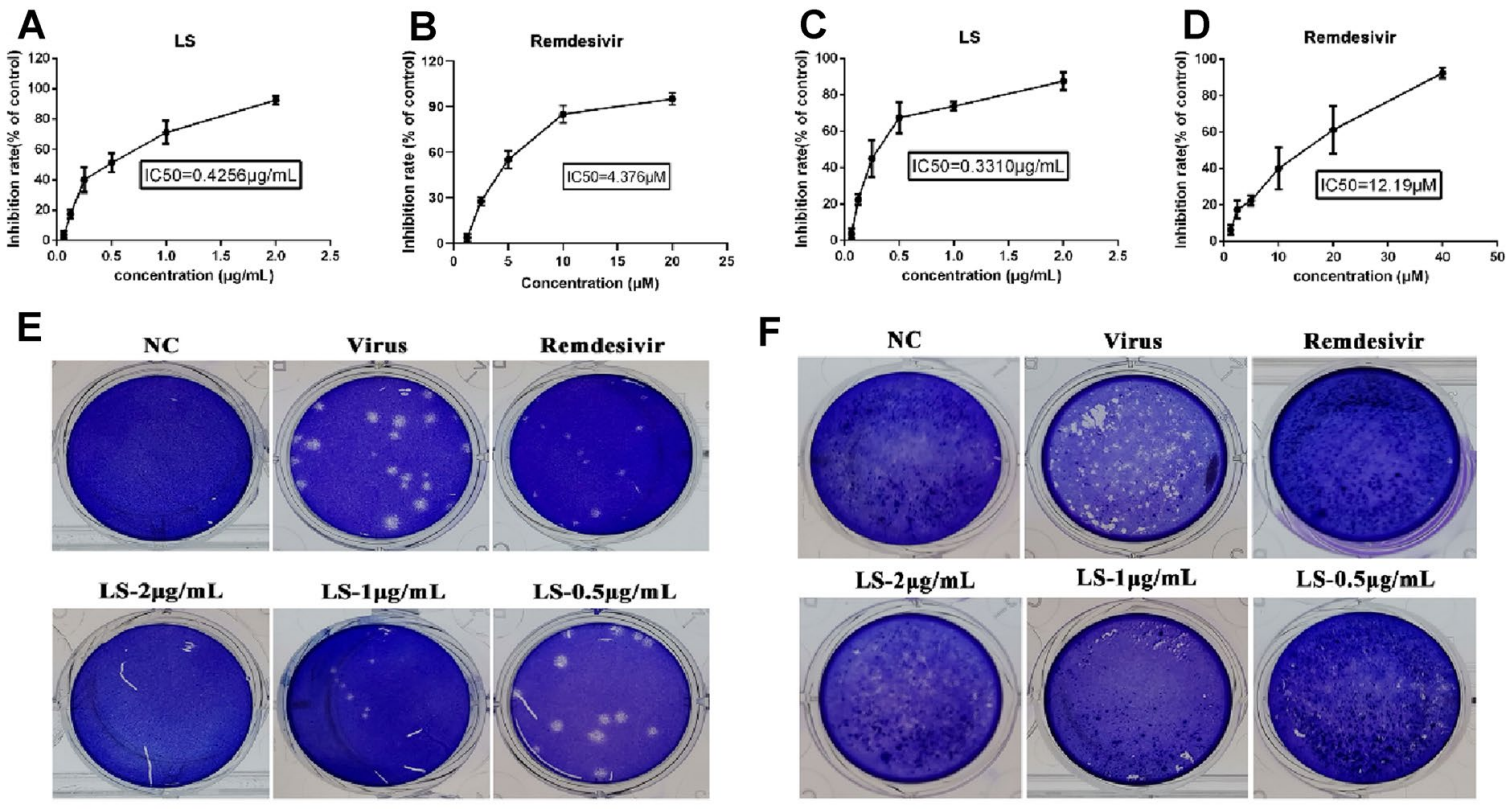
The antiviral activities of LS against 501Y.V2/B.1.35 strain and the G/478K.V1/ B.1.617.2 strain. **A** The inhibitory effects of LS on the 501Y.V2/B.1.35 strain in Vero E6 cells. **B** The inhibitory effects of remdesivir on the 501Y.V2/B.1.35 strain in Vero E6 cells. **C** The inhibitory effects of LS on the G/478K.V1/ B.1.617.2 strain in Vero E6 cells. **D** The inhibitory effects of remdesivir on the G/478K.V1/ B.1.617.2 strain in Vero E6 cells. **E** Inhibitory effect of LS (2 μg/mL, 1 μg/mL and 0.5 μg/mL) on plaque formation of the 501Y.V2/B.1.35 strain. **F** Inhibitory effect of LS (2 μg/mL, 1 μg/mL and 0.5 μg/mL) on plaque formation of the G/478K.V1/ B.1.617.2 strain

The cytotoxic effect of LS on Vero E6 cells has been assessed previously [17]. The TC_50_ of LS toward Vero E6 cells was 4.930 μg/mL. The antiviral activities of LS and remdesivir against the 501Y.V2/B.1.35 strain and G/478 K.V1/ B.1.617.2 strain were evaluated by CPE inhibition assay. As shown in Fig. 1, the results indicated that LS (2 μg/mL, 1 μg/mL and 0.5 μg/mL) significantly reduced the CPE caused by infection in Vero E6 cells. The IC_50_ values of LS and remdesivir were 0.4256 μg/mL and 4.376 μM towad to 501Y.V2/B.1.35 strain (Fig. 1A, B) and the IC_50_ values of LS and remdesivir were 0.331 μg/mL and 12.19 μM towad to G/478K.V1/ B.1.617.2 strain (Fig. 1C, D, respectively. The selectivity index (SI) of LS was 11.58 towad to 501Y.V2/B.1.35 strain and 14.89 towad to G/478K.V1/ B.1.617.2 strain, respectively. Furthermore, the results of plaque reduction assay showed that the plaque number and average size in LS-treated cells were markedly reduced in a dose-dependent manner in the two virus strains (Fig. 1E, F). The CPE assay and plaque reduction assay showed that LS was able to protect cells from virus-induced cell death in a dose-dependent manner and had a board-spectrum of antiviral effects.

### LS strongly inhibits the mRNA expression of pro-inflammatory cytokines induced by the 501Y.V2/B.1.351 strain and G/478 K.V1/ B.1.617.2 strain in vitro

**Fig. 2:**
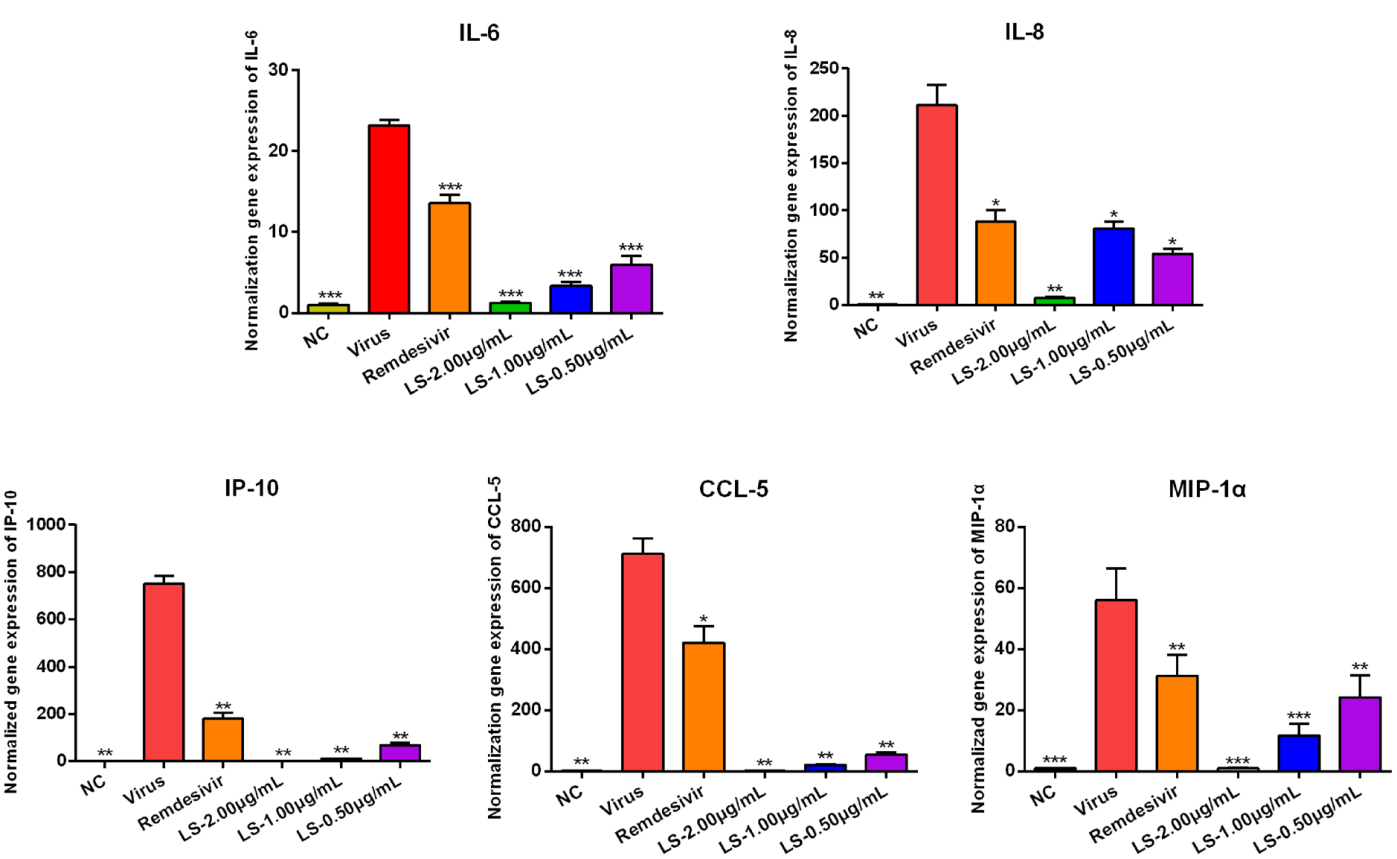
Effects of LS (2, 1 and 0.5 μg/mL) or Remdesivir on the mRNA expression levels of inflammatory mediators (IL-6, IL-8, IP-10, CCL-5 and MIP-1α) in the 501Y.V2/B.1.351 strain-infected cells. Data were presented as the mean ± SD obtained from three separate experiments. *P < 0.05; **P < 0.01; ***P < 0.001, compared with the 501Y.V2/B.1.351 strain-infected cells

**Fig. 3:**
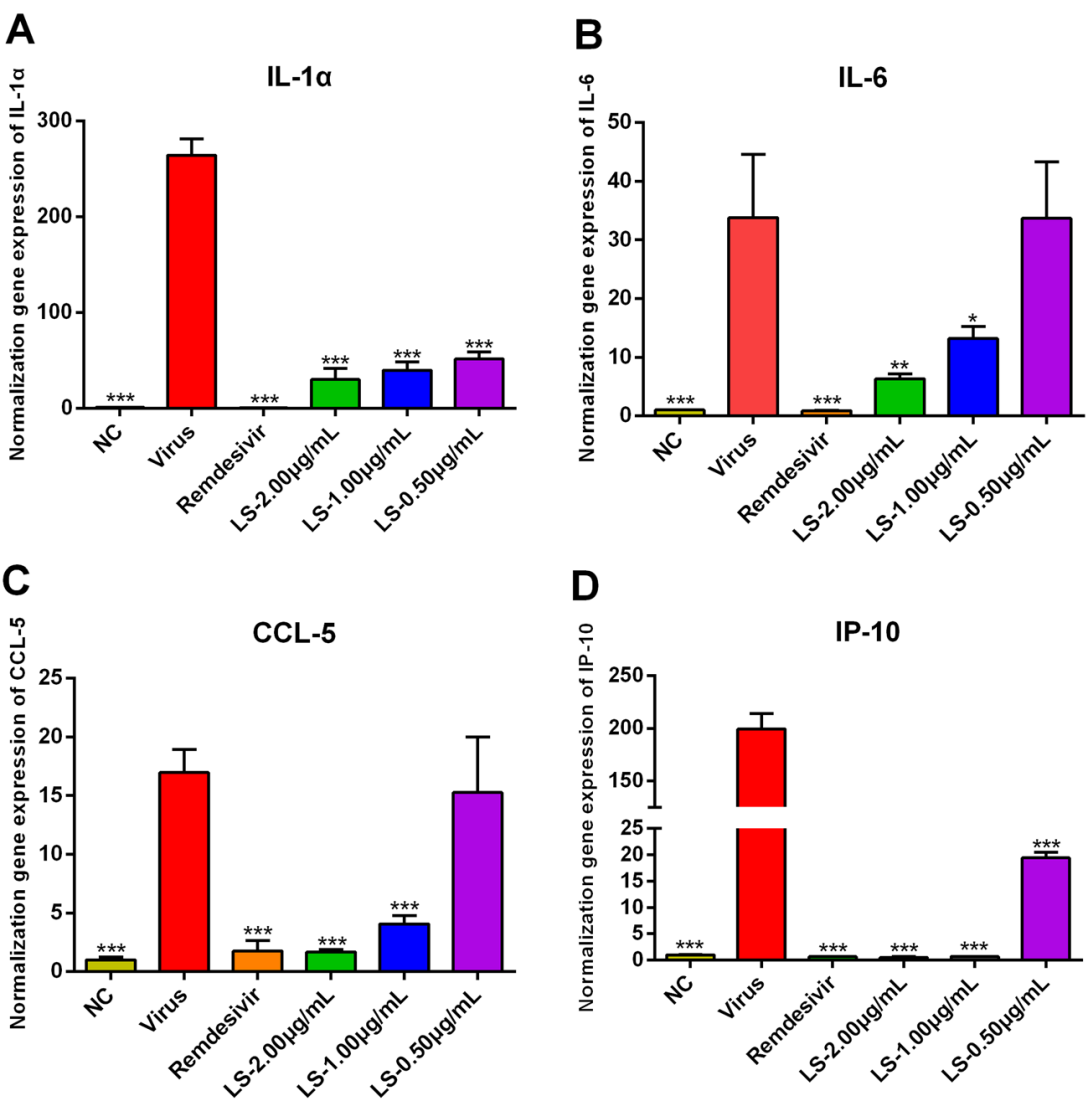
Effects of LS (2, 1 and 0.5 μg/mL) on the mRNA expression levels of inflammatory mediators (IL-1α (**A**), IL-6 (**B**), CCL-5 (**C**) and IP-10 (**D**)) in the G/478K.V1/ B.1.617.2 strain-infected cells. Data were presented as the mean ± SD obtained from three separate experiments. *P < 0.05; **P < 0.01; ***P < 0.001, compared with the G/478K.V1/ B.1.617.2 strain-infected cells

SARS-CoV-2-induced increase of inflammatory cytokines is an important pathological factor in SARS-CoV-2-related acute lung injury (ALI) and ARDS. Under the stimulus of the 501Y.V2/B.1.35 strain infection, the expression of IL-6, IL-8, IP-10, CCL-5, MIP-1α significantly increased after infection for 48 h compared with NC group (*P* < 0.001 or *P* < 0.01). LS (2, 1 and 0.5 μg/mL) could markedly reduce the expression of IL-6, IL-8, IP-10, CCL-5 and MIP-1α in a dose-dependent manner compared with the virus group (*P* < 0.001, *P* < 0.01 or *P* < 0.05) (Fig. 2). To our surprise, the inhibitory effect of LS on these cytokines was better than remdesivir. The same results as the anti-inflammatory on the G/478 K.V1/ B.1.617.2 strain infection. As shown in the Fig. 3, the mRNA expression of IL-6, IP-10, CCL-5 and IL-1α were detected by RT-qPCR. Under the stimulus of the G/478 K.V1/ B.1.617.2 strain infection, the mRNA expression of IL-6, IP-10, CCL-5 and IL-1α significantly was increased after infection for 48 h compared with NC group (P < 0.001). LS (2 μg/mL, 1 μg/mL and 0.5 μg/mL) could markedly reduce the expression of IP-10 and IL-1α compared with the virus group (P < 0.001). LS (2 μg/mL and 1 μg/mL) significantly inhibited the mRNA expression of IL-6 and CCL-5 (P < 0.001, P < 0.01 or P < 0.05). Based on the effects of LS on reduction the pro-imflammatory mediators induced by the 501Y.V2/B.1.351 and the G/478 K.V1/ B.1.617.2 strain, we can conclude that LS is a broad-spectrum anti-inflammatory agent.

### LS significantly improves the survival rate of infected mice, decreases the virus titers of lung and improves the pulmonary histopathological changes of SARS-CoV-2-induced pneumonia mice

**Fig. 4:**
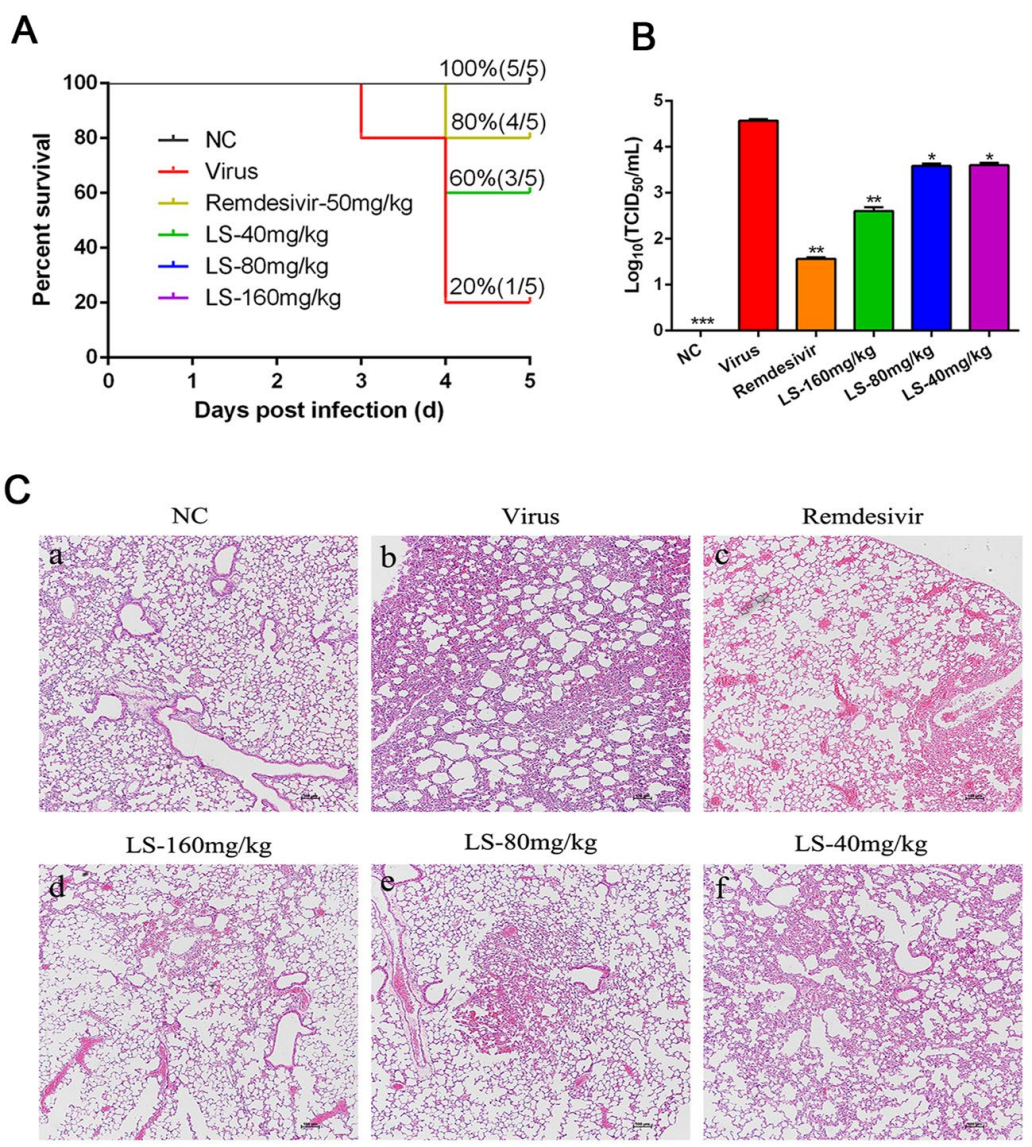
The therapeutic effect of LS or remdesivir on SARS-CoV-2-infected mice. **A** The survival rate was observed for 5 days after viral infection. **B** The pulmonary viral load at the fifth d.p.i. was determined by TCID_50_. **C** Histopathological changes of lung tissues at the fifth d.p.i. Scale bar = 100 µm. **a** Mock infected mice treated with PBS (normal control, NC); **b** SARS-CoV-2-infected mice treated with PBS (viral control, Virus); **c** SARS-CoV-2-infected mice treated with remdesivir (50 mg/kg); **d**–**f** SARS-CoV-2-infected mice treated with LS (160, 80 and 40 mg/kg). *p < 0.05; **p < 0.01; ***p < 0.001, compared with viral control

To future estimate the therapeutic effect of LS, the effect of LS on lethal SARS-CoV-2 infection mice was observed. Mice in the normal group were active, while the infected mice were presented with ruffled fur, huddling, lethargy, and poor appetite. Mice in the model group began to die on day 3 and the survival rate was 20% (Fig. 4A). The survival rate of mice treatment with LS (160 and 80 mg/kg) were 100% for 5 days observation (Fig. 4A). Treatment with LS (40 mg/kg) and remdesivir (50 mg/kg) could also increase the survival time and the survival rate of them was 60 and 80%, respectively (Fig. 4A). These results indicated that treatment with LS could significantly improve the mortality and extend the life span of infected mice.

To evaluate the effect of LS on resisting virus in mice, the virus titers in the lung was determined. Compared with the normal group, the virus titers of the model group were markedly increased (Fig. 4B) (*P* < 0.001). Treatment with LS (160, 80 and 40 mg/kg) and remdesivir (50 mg/kg) could decrease the virus titers (Fig. 4B) (*P* < 0.01 or *P* < 0.05).

To determine the effect of LS on SARS-CoV-2-induced pneumonia, lung histological of each group was observed. No histopathological change was observed in the normal group (Fig. 4Ca). While the lung of infected mice showed the presence of interstitial edema, thickened alveolar walls, infiltration of inflammatory cells and alveolar hemorrhage (Fig. 4Cb). Treatment with LS (160, 80  and 40 mg/kg) and remdesivir (50 mg/kg) could improve SARS-CoV-2-induced pulmonary histopathological changes and decrease the destruction of alveolar wall and alveolar exudation (Fig. 4Cc–f). These results indicated that LS could sign.

### LS strongly inhibits SARS-CoV-2-induced production of cytokines and the expression of the key proteins related to the NF-κB/MAPK signaling pathway in vivo

**Fig. 5:**
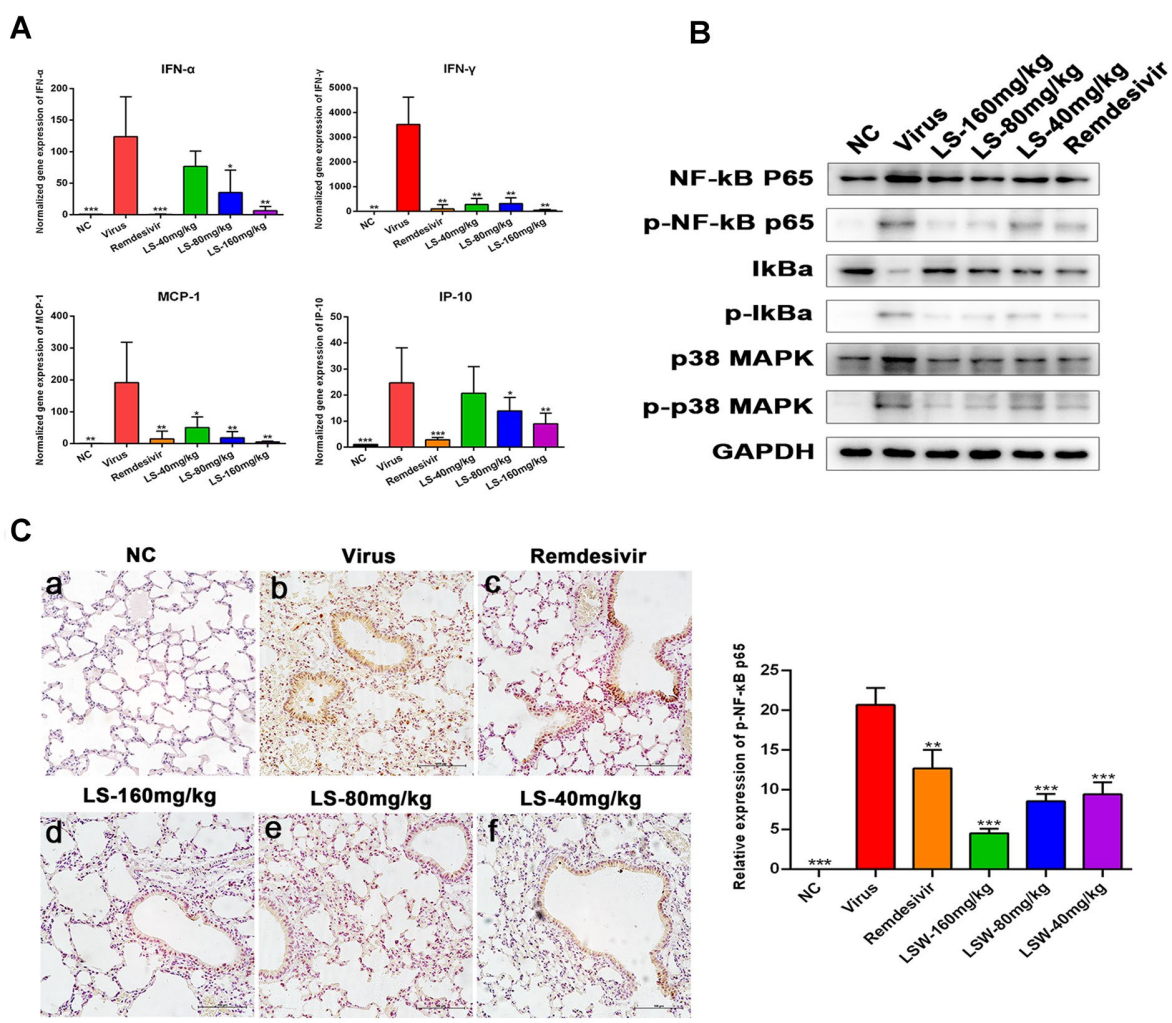
Effects of LS on the expression levels of inflammatory mediators and the expression of the key proteins related to the NF-κB/MAPK signaling pathway in SARS-CoV-2-infected mice. **A** The mRNA expression of the IFN-α, IFN-γ, MCP-1 and IP-10 in the lung of the infected mice were detected by RT-qPCR analysis at the fifth d.p.i.; **B** The expression of the key proteins related to the NF-κB/MAPK signaling pathway was examined by western blotting; **C** The expression of phosphorylation of nuclear translocation of NF-κB p65 was determined by immunohistochemistry at the fifth d.p.i. Scale bar = 400 µm. **a** mock infected mice treated with PBS (normal control, NC); **b** SARS-CoV-2-infected mice treated with PBS (viral control, Virus); **c** SARS-CoV-2-infected mice treated with remdesivir; **d**–**f** SARS-CoV-2-infected mice treated with LS (160, 80 and 40 mg/kg). *P < 0.05; **P < 0.01; ***P < 0.001, compared with viral control

Interferon and Chemokines can be activated by SARS-CoV-2 in COVID-19, which plays an important role in the lung pathology of COVID-19. Thus, the production of cytokines in mice were determined. Compared with the normal group, the mRNA expression of IP-10, MCP-1, IFN-α and IFN-γ in the infected mice were significantly elevated (*P* < 0.001 or *P* < 0.01). Treatment with LS (160 and 80 mg/kg) could effectively reduce the expression of IP-10 and IFN-α (*P* < 0.01 or *P* < 0.05) (Fig. 5A). Treatment with LS (160 and 80 mg/kg) and remdesivirsignificantly inhibited the mRNA expression of IFN-α, MCP-1, IP-10 and IFN-γ (*P* < 0.01 or *P* < 0.05), and treatment with LS (40 mg/kg) significantly inhibited the mRNA expression of MCP-1 and IFN-γ (*P* < 0.01 or *P* < 0.05).

To further study whether the antiviral and anti-inflammation mechanisms induced by virus of LS was related to the inhibitory of NF-κB/MAPK signaling pathway activation, the expression of the key proteins related to the NF-κB/MAPK signaling pathway was examined by western blotting and immunohistochemistry(Fig. 5B, C). The results showed that the protein expressions of p-NF-κB p65, p-IκBα, and p-p38 MAPK of the virus group were significantly increased compared with the NC group (*p* < 0.05), and the expression of IκBα of the virus group were significantly decreased (*p* < 0.05). Compared with the virus group, the protein expression of the p-NF-κB p65, p-p38 MAPK and p-IκBα were significantly reduced in the group treated with (160, 80 and 40 mg/kg), and IκBα was significantly upregulated, while the expression of NF-κB p65 and p38 MAPK was no significant difference. Morrover, the phosphorylations of nuclear translocation of NF-κB p65 in SARS-CoV-2-infected mice was determined by immumohistochemical staining. The result also showed that there was a lower expression of p-NF-κB p65 in the group treated with (160, 80 and 40 mg/kg) when compared with the model group (*P* < 0.001 or *P* < 0.01). The effect of LS on inhibition of p-NF-κB p65 was superior to remdesivir. These results showed that LS could reduce the expression of cytokines in the lung probably via interruption of NF-κB/MAPK signaling pathway.

### LS significantly accelerates recovery, ameliorates symptoms and shortens the duration of viral shedding in patient

Patient A was presented with dry cough and was included On March 17, 2020. The time to conversion to nucleic acid negativity lasted for 40 days. After receiving treatment for 56 days, patient A completely relieved his symptoms and was discharged. Patient B was recruited On March 28, 2020, who was presented with dry cough as well, but dry cough was immediately disappeared after being treated for 6 days which was markedly shorter than patient A. Patient B had shorter length of hospital stay and was discharged after being included in the study for 23 days with negative nucleic acid test twice On April 19, 2020 and April 20, 2020. Specifically, treatment with LS and was associated with accelerated symptom resolution and rapid SARS-CoV-2 clearance. These results indicated that LS could effectively improve prognosis of patient in relieving symptoms and resisting virus.

### LS suppresses excessive release of inflammatory meditors in patient B

During treatment, the protein expression of inflammatory factors in both patients were monitored. There was a significant increase in serum inflammatory factors (PDGF-AA/BB, Eotaxin, MCP-1, MIP-1α, MIP-1β, GRO, CCL-5, MCP-3, IL-6, IP-10, IL-1α) of patient A. Conversely, the inflammatory factors of patient B remained stable even lower. These results indicated that treatment with LS could prevent patient from the hyperinflammatory status.

### Safety evaluation

No adverse events were observed in patient treated with LS and no reports of abnormal laboratory blood tests were reported. The indexes related to liver function (ALT, AST, GGT, TBIL, DBIL, TP, ALB and TBA), renal function (K, Na, Cl, TCO_2_, Ca, GLU, UREA, CCr and CREA) and cardiac function (CK, CK-MB, LDH and HBDH) were normal (Table 3).

**Table 3:** Biochemical indexes for safety evaluation

Biochemical indexes	Patient A		Patient B	
	Day1	Day7	Day1	Day7
ALT(IU/L)	13.3	15.8	14.1	6.7
AST(IU/L)	13.6	15.0	12.9	18.1
GGT(IU/L)	19.3	17.1	83.7	45.5
TBIL(μmol/L)	11.9	18.4	10.4	11.5
DBIL(μmol/L)	2.2	4.0	1.6	2.0
TP(g/L)	72.2	65.9	67.1	69.3
ALB(g/L)	45.1	42.0	41.0	42.6
TBA(μmol/L)	6.3	16.2	4.7	8.9
CCr(mL/min)	114	116.3	84.2	85.6
CREA(μmol/L)	63.7	62.4	94.10	96
UREA(μmol/L)	6.3	3.8	5.8	5.7
K(μmol/L)	3.84	3.77	4.29	3.99
Na(μmol/L)	138.8	140.2	141.9	142.3
Cl(μmol/L)	106.7	107.1	106.8	104.0
TCO_2_(μmol/L)	22.5	25.6	26.5	29.7
Ca(μmol/L)	2.28	2.40	2.30	2.33
GLU(μmol/L)	5.40	7.66	5.25	5.29
LDH(IU/L)	151.5	139	187.4	162.3
CK(IU/L)	60.8	61.4	31.3	40.1
CK-MB(IU/L)	12.0	12.0	5.0	3.0
HBDH(IU/L)	104.0	95.0	120.0	109.0

*ALT* Alanine Aminotransferase, *AST* Aspartate Aminotransferase, *GGT* γ-glutamyl transpeptidase, *TBIL* Total Bilirubin, *DBIL* Direct Bilirubin, *TP* Total Protein, *ALB* albumin; *TBA* Total Bile Acids, *CCr* Creatinine Clearance, *CREA* creatinine, *K* Potassium ions, *Na* Sodium ion, *Cl* Chloride ion, *TCO2* Total carbon dioxide, *Ca* Calcium ions, *GLU* Glucose; *LDH* Lactic Dehydrogenase, *CK* Creatine kinase, *CK-MB* creatine kinase-MB, *HBDH* hydroxybutyrate-dehydrogenase

## Discussion

COVID-19, an acute respiratory tract infection caused by SARS-CoV-2, not only causes various respiratory symptoms but also elicits pulmonary inflammation and infiltration even induces a strong cytokine storm, leading to systemic inflammatory response and multiple organ failure. In addition, SARS-CoV-2 is very susceptible to mutation and the variants have spread around the world. Public health and social measures (PHSM) recommended by WHO have been effective in reducing COVID-19 cases, hospitalizations and deaths. However, there is a decrease in effectiveness of vaccines [22] and drug therapeutics [23], making a challenge for the prevention and treatment for the SARS-CoV-2 variants. LS is a Chinese patent medicine with anti-inflammatory and antiviral effects and has been widely utilized in clinical treatment of acute pharyngitis [24], tonsillitis [25], acute bronchitis [26] and influenza infection [27] in China. Our previous study had found that LS had antiviral and anti-inflammatory activities against SARS-CoV-2 in vitro [17]. In the present study, we further studied its antiviral and anti-inflammatory activities against variants including the 501Y.V2/B.1.35 and G/478 K.V1/ B.1.617.2 strains in vitro, verified its effect on SARS-CoV-2 in vivo and investigated its efficacy and safety in COVID-19.

As of 14 September 2021, SARS-CoV-2 variants of Concern Alpha, Beta, Gamma and Delta have been prevalent in 193, 142, 96 and 180 countries, respectively [28]. To our surprise, the basic reproduction number (R0) of the Delta variant (5.08) is much higher than the R0 of the ancestral strain of 2.79 [29]. According to a recent study, the 501Y.V2/B.1.35 strain not only disables three classes of monoclonal antibodies, but it also evades the neutralizing antibodies from the serum of recovered COVID-19 [3]. Inhibition of viral cytopathic effect (CPE) is a classical in vitro antiviral assays via determining visually and inhibition of viral plaque formation is a assay to evalute the effect of LS on virus yield [30]. Our results indicated that LS could effectively inhibit the proliferation of the 501Y.V2/B.1.35 strain and suppress the excessive release of mediators induced by it in vitro. Studies had indicated that the G/478K.V1/ B.1.617.2 strain could increase its ability to spread and replicate in vivo and enhance its infectivity comepared with the wild-type strain in vitro [31, 32]. Interestingly, compared with the inhibitory effects of remdesivir on the 501Y.V2/B.1.35 strain and the novel SARS-CoV-2, the IC_50_ of remdesivir against the G/478 K.V1/B.1.617.2 strain (12.19 μM) was higher than the 501Y.V2/B.1.35 strain (4.376 μM) and the novel SARS-CoV-2 (0.6505 μM) [17]. While our results showed that the IC_50_ of LS againt the G/478 K.V1/ B.1.617.2, 501Y.V2/B.1.35 and the novel SARS-CoV-2 was 0.331 μg/mL, 0.4256 μg/mL and 0.6024 μg/m [17], respectively. Therefore, LS posed broad-spectrum antiviral activity against SARS-CoV-2 and the variants. Similarly, LS also could signiciantly inhibit the mRNA expression of inflammatory factors (IL-6, IP-10, CCL-5, IL-1α) induced by the G/478 K.V1/ B.1.617.2 strain. Therefore, we can conclude that LS is a broad-spectrum anti-inflammatory agent, which can inhibit the excessively secreted pro-inflammatory cytokines induced by SARS-CoV-2 and viriants probably via regulating the NF-κB signaling pathway.

Our previous study has indicated that LS posed anti-viral and anti-inflammatory activities against SARS-CoV-2 in vitro. Cell-based antiviral assays can quickly determine the effects of LS on SARS-CoV-2. But the results of cell-based assays can’t fully reflect the effects of agents in vivo. The animal models play a vital role in evaluating novel therapies and vaccines. Nowadays, most of the mouse models have low lethality and vary in disease severity [33]. The Ad5-hACE2-transduced mice did not cause severe disease and extrapulmonary manifestations of disease [34]. Typical histopathology of lung, weight loss, virus replication and upregulated innate immune response have been observed in human ACE2-transgenic mouse models [35, 36]. However, only K18-hACE2 mice could develop severe disease and cause high lethality [36, 37]. Mouse models with mild and severe disease could fully reflect mild and severe COVID-19, which could be ultilized for studies of pathogenesis of COVID-19 and evaluation of antiviral drugs and vaccines. Thus, we established the lethal and acute pneumonia K18-hACE2 transgenic mice models of SARS-CoV-2 for further validation of the therapeutic effects of LS. Our results showed that the mortality rate of mice in the model group was 80% on the fifth day after infection. LS could significiantly reduce mortality and prolong survival time. The pulmonary pathology indicated that SARS-CoV-2 could lead to significant alveolar wall thickening, interstitial edema, immune cell infiltration and lung consolidation. Lung pathology of infected mice treated with LS was markedly alleviated. Viral replication in the lung can be followed by immune response including immune cell infiltration and release of pro-inflammatory cytokines, which can contribute to severe lung pathology of mice [36]. When treated with LS, lung virus titers of infected mice were significiantly reduced. In line with the effect of LS on inhibition of pro-inflammatory mediators in patient, LS could significantly reduce the mRNA expression of cytokines (IFN-α, IFN-γ, MCP-1 and IP-10) in the lung of infected mice as well. Our previous study has found that LS could inhibit the exuberant expression of cytokines induced by SARS-CoV-2 through NF-κB signaling pathway in vitro. NF-κB signaling pathway can be triggered by respiratory pathogens and blocking the NF-κB pathway has been considered as a potent strategy in influenza treatment [38]. In addition, NF-κB signaling pathway is a central cellular pro-inflammatory signal pathway and NF-κB hyper-activation can cascade of pro-inflammatory cytokines and chemokines [39], which was considered to be a key pathway of ALI 40]. A recent study had indicated that SARS-CoV-2 could induce NF-κB hyper-activation and pro-inflammatory responses [41] and NF-κB signaling pathway has been considered as a promising treatment target for COVID-19 [39, 42, 43]. Similarly, our finding indicated that LS could significantly decrease SARS-CoV-2-induced activation of p-NF-κB p65, p-IκBα, and p-p38 MAPK and increase the expression of the IκBα by western blotting and LS could inhibit the phosphorylations of nuclear translocation of NF-κB p65 by immumohistochemical staining. These resuts indicated that the underlying mechanism of LS was to impair the upregulated proinflammatory cytokines induced by SARS-CoV-2 via inhibiting the activity of NF-кB/MAPK signaling pathway.

In order to further confirm the anti-novel coronavirus effects of LS on COVID-19, two cases of novel Coronavirus pneumonia were observed in the next study. Negnitive PCR-proven test is the key for discharge from the hospital. A study indicated that the median time from symptom onset to viral clearance on real-time RT-PCR was 34 days [44]. Our results showed that the time to conversion to nucleic acid negativity was 23 days and the time from symptom onset to symptom disappearing was 6 days in the LS-treatment patient, which indicated that LS could effectively improve the disease prognosis compared with the non-LS-treatment group. The aberrantly activated cytokines in the lung could result in diffuse alveolar damage, alveolar edema and proteinaceous exudates, thickening of alveolar walls, evident desquamation of pneumocytes and hyaline membrane formation [45]. The aberrant cytokines in the lung also play a central role in severity and lethality in various acute respiratory viral infections including Influenza A (H5N1), highly pathogenic H1N1, SARS-CoV, MERS-CoV and SARS-CoV-2. A variety of cytokines and chemokines including IL-1α, IL-6, IP-10, MCP-1, MIP-1α and IFN-γ had been found to be significantly elevated in the serum of COVID-19 patients [46, 47]. IL-6, MCP-1 and IP-10 have been found to be strongly associated with disease severity in COVID-19 [48, 49]. If not properly treated, the exuberant and prolonged cytokines with the rapid viral replication would lead to ALI, ARDS, multiorgan failure, even death [50]. Specific cytokine inhibitors such as tocilizumab and had been used as treatment on COVID-19 against the consequences of the elevated levels of cytokines [51]. Our clinical obeservation indicated that the patient B showed low level of cytokines such as IL-6, MCP-3, MCP-1, IP-10 and IL-1α after treatment with LS, which hinted that LS could prevent COVID-19 into hyperinflammatory status. Though only one patient was recruited, this preliminary clinical observation had indicated that LS had great potential to exert therapeutic effect on COVID-19 and these results provided a theoretical foundation for clinical applications.

## Conclusion

In conclusion, LS significantly inhibited virus replication of the variants and pro-inflammatory factors induced by the variants in vitro, effectively alleviated SARS-CoV-2-induced ALI in mice and had clinical advantages for COVID-19. The therapeutic effects of LS were attributed to inhibition of proliferation of virus and anti-inflammatory effects possibly via blocking the NF-кB/MAPK signaling pathway. These results provide more evidence for the treatment of LS for COVID-19 and reveal the potential mechanism of LS against SARS-CoV-2. Therefore, LS is expected to be an effective therapeutic agent for COVID-19. To provide a strong rationale for their use on COVID-19, a large-scale randomized, double-blinded, placebo controlled clinical trial is necessary to be carried out to confirm the effect of LS on COVID-19.

## Data Availability

The datasets in this study are available from the first author and the corresponding author on reasonable request.

## Abbreviations

TCM: Traditional Chinese Medicine
LS: Liushen Capsules
MCP-1: Macrophage chemoattractant protein-1
MIP-1β: Macrophage inflammatory protein-1β
IL-6: Interleukin-6
IP-10: Interferon-inducibleprotein10
sCD40L: Soluble CD40 ligand
TCID_50_: 50% Tissue culture infective dose
cytopathic effect: CPE
IC_50_: The 50% inhibition concentration
SI: Selectivity index
TC_50_: Median toxic concentration
PFU: Plaque-forming unit
IFN-α: Interferon-α
IFN-γ: Interferon-γ
PDGF-AA/BB: Platelet derived growth factor-AA/BB
MIP-1α: Macrophage inflammatory protein-1α
GRO: Growth-related oncogene
MCP-3: Macrophage chemoattractant protein-3
IL-1α: Interleukin-1α
NF-κB: Nuclear factor-kappa B
CCL-5: Chemokine ligand 5
ALI: Acute lung injury
IκBα: Inhibitor of NF-κB
MAPK: Mitogen-activated protein kinase
